# The relationship between mobile addiction and nomophobia: What role does age play?

**DOI:** 10.3389/fpsyg.2026.1706449

**Published:** 2026-05-07

**Authors:** Victoria García, Mehran Mohebi, Beatriz Sora, María José Serrano-Fernández, Christine Unterrainer

**Affiliations:** 1 Department of Psychology, Universitat Rovira i Virgili, Tarragona, Spain; 2 Department of Applied Psychology, Universität Innsbruck, Innsbruck, Austria

**Keywords:** age, mobile addiction, nomophobia, psychological problem, smartphone

## Abstract

**Background:**

Over recent decades, smartphones have undoubtedly meant great progress for contemporary society and have changed our daily lives. They have involved not only benefits but also some problems, such as nomophobia and mobile addiction. Nomophobia represents the fear of being unreachable via one’s smartphone; whereas mobile addiction is understood as chronic or periodic obsessions with mobile use, which may lead to intense and sustained demand and reliance. Although they are different phenomena, they have been interchangeably used in the research, and their potential relationship has been unexplored. Accordingly, the present study aims to clarify the relationship (and overlap) between nomophobia and mobile addiction, and also to provide additional evidence on the possible intervening factors in these phenomena and their relationship, such as age.

**Method:**

The opportunity sampling was used to collect the sample. Spanish workers (*n* = 366) participated in this study. 44.54% were men (*n* = 163) and 55.46% women (*n* = 203). Their ages ranged from 18 to 99 years (mean = 40.43; SD = 13.31).

**Results:**

The concepts of mobile addiction and nomophobia are capable of capturing a pathological relationship between people and their smartphones. The results showed differences in nomophobia and mobile addiction levels between older and younger people. The latter being the ones that present a more problematic use of the mobile, since it is the generation that has grown up together with technology. Finally, age played a moderator role in the relationship between both phenomena.

**Conclusion:**

Our study evidenced that age is a critical factor for nomophobia and mobile addiction and their relationship. Finally, it is critical to study them as distinct but related constructs to advance in their understanding in future research.

## 1 Introduction

The appearance of information and communications technology has meant a great leap forward for modern life. Nevertheless, unhealthy and/or harmful use can also have negative consequences, including psychological problems related to smartphone use, such as nomophobia and mobile addiction (Rosales-Huamani et al., 2019; Tran, 2016). Nomophobia, an abbreviation of “no mobile phone phobia,” is the “irrational” fear of not being able to use one’s smartphone (Argumosa-Villar et al., 2017; King et al., 2013; King et al., 2014; Yildirim and Correia, 2015), and more specifically, the fear of not being able to communicate, of losing the connectedness that smartphones provide, of not being able to access information, and of giving up convenience (Yildirim and Correia, 2015). Mobile addiction is a kind of technology addiction characterized by symptoms such as conflict, withdrawal, relapse, and behavioral salience (Soror et al., 2015). Although these constructs are widely studied in the literature, neither nomophobia nor mobile addiction is currently recognized as a formal psychiatric diagnosis, and they are better understood as behavioral or psychological patterns associated with problematic smartphone use (Kaviani et al., 2020; Humood et al., 2021).

Although phobia and addiction are clearly differentiated in the psychology literature, there is an overlap between the constructs of nomophobia and mobile addiction. Indeed, they have been used interchangeably in the research (e.g., Güzel, 2018; Argumosa-Villar et al., 2017; Myakal and Vedpathak, 2019). Some theorists argue that nomophobia is a misused term and can be better interpreted as a type of anxiety, addiction, or behavioral disorder rather than proper fear (Bianchi and Phillips, 2005; Bragazzi and Del Puente, 2014). Therefore, the way in which problematic use is defined, measured, and contextualized is a matter of significant debate (Kaviani et al., 2020; Billieux et al., 2008; Lopez-Fernandez et al., 2014), because the variability in how they are conceptualized may have a critical impact on how they are understood. It is also necessary to better understand the relationship between smartphone addiction and nomophobia. Little research has examined these disorders from the point of view of being different constructs that may be related. In addition, these psychological disorders seem not to affect everyone in the same way. A large segment of the literature has looked at them in samples of younger people by assuming that the use of smartphones with technical innovations is more common at this stage of life (e.g., Ozkan and Solmaz, 2015; Chóliz, 2012; Kanmani et al., 2017; Durak, 2019). A smaller group of empirical studies has shown that younger users experience higher levels of nomophobia and mobile addiction (e.g., Shankar et al., 2018), but it is also possible to find studies that present non-significant differences in the function of age (e.g., Moreno-Guerrero et al., 2020). Most of this limited source of empirical research has mainly provided support through descriptive analysis (e.g., Shankar et al., 2018). It is therefore necessary to provide empirical evidence for this assumption and examine the differences at various age stages, especially regarding the relationship between mobile addiction and nomophobia. Thus, the present study aims, on one hand, to clarify the relationship (and overlap) between mobile addiction and nomophobia, and on the other to explore the role of age in this relationship.

In the literature, there is a conceptual vagueness about the construct of nomophobia insofar as it has been used indiscriminately to refer to phobias and mobile addiction disorders (Durak, 2019; Davie and Hilber, 2017). This means we can find studies that have conceptualized nomophobia as a phobia (Bragazzi and Del Puente, 2014; Przybylski et al., 2013; Bhattacharya et al., 2019; Gezgin and Çakır, 2016; Apak and Yaman, 2019; Tams et al., 2018) along with others that have understood it as a mobile addiction (e.g., Basu et al., 2018; Sood and Butt, 2020; Rodríguez-García et al., 2020; Aznar-Díaz et al., 2019; Siddiqi et al., 2017).

Undoubtedly, the main reason for this confusion is that nomophobia and mobile addiction share many qualities (Tran, 2016).

However, the psychology literature provides evidence that nomophobia and mobile addiction should be considered distinct but related constructs. According to the DSM (Diagnostic and Statistical Manual of Mental Disorders), phobias are characterized by being intense, irrational fears with no apparent foundation to explain the symptoms, anxiety, and panic. A specific phobia, such as nomophobia, represents the fear generated by a lack of access to a mobile phone. In contrast, addiction can be described as an unusually high dependence on a particular medium, such as a smartphone (Chiu, 2014). Hanley and Wilhelm (1992) and Park (2005) define addictive behavior as any activity, substance, object, or behavior that has become the major focus of a person’s life to the exclusion of other activities. In this vein, the research has also started to point out that nomophobia and mobile addiction must be considered as different disorders (Buctot et al., 2020). This assumption is underpinned according to how users behave with their smartphones. A user can suffer from nomophobia if they present fear or anxiety in connection with not using a smartphone (Bian and Leung, 2015; Emanuel et al., 2015; SecurEnvoy, 2012; Yildirim and Correia, 2015), whereas a user may have a mobile addiction when they use a smartphone to excess regardless of the harmful consequences (Bian and Leung, 2015). These disorders also seem to present differences in their detrimental outcomes. Studies that have measured nomophobia as a mobile addiction show a stronger relationship with anxiety (Anshari et al., 2019; Gezgin and Çakır, 2016), while those that have measured it as a phobia present a stronger relationship with depression than with anxiety (Arpaci, 2020).

Although nomophobia and mobile addiction do appear to be different, they cannot be considered to be unrelated disorders (Tran, 2016). As Yildiz et al. (2019, p.7) has explained, “like with individuals who have not been able to control their concerns about staying away from smartphone use seem to have used their phones problematically. For this reason, it is logical to argue that smartphone addiction is related to nomophobia” (Durak, 2018). Research suggests that mobile addiction can be a critical determinant of nomophobia (Bhattacharya et al., 2019; Pavithra et al., 2015). Although several studies have reported positive associations between mobile addiction and nomophobia, including recent work using more advanced psychometric approaches (e.g., Ren et al., 2024), important questions remain regarding the contextual and developmental factors that may influence this relationship. Indeed, we are aware of only one study, that by Yildiz et al. (2019), which has empirically examined this association and shown a positive correlation between nomophobia and mobile addiction. The author concluded that those individuals at risk of mobile addiction are also highly likely to become nomophobic. Studies can also be found that show this relationship moving in the opposite direction, i.e., presenting nomophobia as the predictor of mobile addiction (Semerci, 2019). Although previous research has frequently reported higher levels of problematic smartphone use among younger individuals (e.g., Salehan and Negahban, 2013), important gaps remain in understanding the mechanisms underlying these differences. Much of the existing literature has relied on student samples or has treated nomophobia and mobile addiction as interchangeable constructs (Billieux et al., 2015). Recent studies, such as Elhai et al. (2017) and Sui and Sui (2021), have pointed out that this conceptual overlap has led to confusion about the true nature of these phenomena. Consequently, it remains unclear whether these phenomena represent distinct but related processes across adulthood and whether age influences not only their levels but also the strength of their association. Addressing these issues is important for clarifying theoretical models of problematic smartphone use and identifying potential vulnerability patterns across the lifespan. Therefore, the present study contributes to the literature by simultaneously examining (a) the conceptual distinction between mobile addiction and nomophobia, (b) their association across multiple dimensions of nomophobia, and (c) the moderating role of age in a heterogeneous adult working sample spanning a wide age range. Bearing all this in mind, we hypothesize that:

*Hypothesis 1:* Mobile addiction is positively related to nomophobia: the fear of not being able to communicate by smartphone (H1a), of losing the connectedness that smartphones provide (H1b), of not being able to access information (H1c), and of giving up convenience (H1d).

In the digital age, consuming and being are inseparable concepts. People seek, express, confirm, and determine their being through what they consume. It is therefore not surprising that separation from the device represents a reduction of oneself, causes anxiety, irritability, and other symptoms similar to those of withdrawal from addictive substances.

Mobile phones have become an important part of our techno culture, especially among the younger population. They have many attributes and characteristics that make them particularly attractive and encourage their use among young people. In fact, they have different functions (Chóliz, 2012): (1) they strengthen personal autonomy, (2) they provide identity and prestige in the context of peer relationships, (3) they offer major technological innovations, tools for which the young show a special preference and skills, (4) they are a source of fun and entertainment, and (5) they help establish and maintain interpersonal relationships. Young people have therefore adopted these devices as an integral part of their daily lives, having them constantly in their awareness, thinking about them when not using them, and being distracted from other tasks when they have their phone with them (Walsh and White, 2006; Walsh et al., 2008).

However, the uncontrolled or inappropriate use of mobile phones can give rise to serious health and social problems (Chóliz, 2012; Van Velthoven et al., 2018; Wacks and Weinstein, 2021; Kim et al., 2015). Younger people spend significantly more time using smartphones compared to older people, and this may lead to problematic involvement and dangerous use (Chiu, 2014) and eventually to psychological disorders such as mobile addiction or nomophobia. Many studies in the literature suggest that mobile addiction and nomophobia are more common among adolescents and youngsters than among other age groups (Aggarwal, 2013; Gezgin and Çakır, 2016; Netburn, 2012; Alavi et al., 2014; Wang et al., 2017). The SecurEnvoy study (SecurEnvoy, 2012), for example, found that young adults are more likely to be mobile addicted compared to older people. It described how most teens (77%) reported anxiety when they were without their mobile phones.

Taking this incongruence into account, the effect of age on nomophobia and mobile addiction needs to be better understood. Following the study by Argumosa-Villar et al. (2017), we would suggest that younger people are more likely to show problematic behaviors when using mobile phones because they are more vulnerable, given that they are not yet in full control of their impulses and accept the use of mobile phones as a status symbol. As far as the relationship between mobile addiction and nomophobia is concerned, we would argue that younger people who present mobile addiction will be more likely to experience nomophobia compared to older people. Bearing all this in mind, we now also propose the following hypotheses:

*Hypothesis 2*: There are different levels of nomophobia in function of age. Compared to older people, younger people will present levels of nomophobia to a higher degree: the fear of not being able to communicate by smartphone (H2a), of losing the connectedness that smartphones provide (H2b), of not being able to access information (H2c), and of giving up convenience (H2d).

*Hypothesis 3*: There are different levels of smartphone mobile addiction in function of age. Younger people will present higher levels of mobile addiction compared to older people.

*Hypothesis 4*: Age modulates the relationship between mobile addiction and nomophobia: the fear of not being able to communicate by smartphone (H4a), of losing the connectedness that smartphones provide (H4b), of not being able to access information (H4c), and of giving up convenience (H4d).

## 2 Method

### 2.1 Procedure

Opportunity sampling, also known as accidental random sampling, was used to collect the sample. The individuals were recruited in the streets and were asked to participate voluntarily after being informed about the research project and its objectives. Most of them agreed to collaborate. Once they had joined the study, with their consent, they were asked to fill in the questionnaires. They completed the questionnaires by hand, returning them in person. Anonymity was guaranteed, and they were assured that all data would remain confidential. The study was conducted in accordance with the Declaration of Helsinki, and the protocol followed the guidelines of the Ethics Committee of our university (CEIPSA-2023-TDO-0002).

### 2.2 Participants

The sample was made up of 366 workers from Tarragona (Spain); 44.54% were men (*n* = 163) and 55.46% women (*n* = 203). Their ages ranged from 18 to 99 years (mean = 40.43; SD = 13.318); 55.5% were married (*n* = 203), 35% single (*n* = 128), 8.5% divorced (*n* = 31) or separated, and 1.1% widowed (*n* = 4). Regarding education, 1.4% of the total sample had no studies (*n* = 5), 7.9% had completed primary studies (*n* = 29), 57.9% had secondary studies (*n* = 212), 22.1% had university studies (*n* = 81), and 10.7% had post-graduate studies (*n* = 39).

### 2.3 Measures

#### 2.3.1 Age and gender

Age was requested in years, and gender was recorded as male (1) or female (2).

#### 2.3.2 The nomophobia questionnaire

The Nomophobia Questionnaire (NMP-Q; Yildirim and Correia, 2015) consists of 20 items with a 7-point Likert scale ranging from 1 “Strongly disagree” to 7 “Strongly agree”. It has four dimensions: (1) fear of not being able to communicate (6 items; example item 12: “I would feel nervous because I would not be able to receive text messages and calls”; Alpha = 0.94); (2) losing the connectedness that smartphones provide (5 items; example item 17: “I would be uncomfortable because I could not stay up-to-date with social media and online networks,” Alpha = 0.94); (3) not being able to access information (5 items; example item 4: “I would be annoyed if I could not use my smartphone and/or its capabilities when I wanted to do so,” Alpha = 0.94); and (4) giving up convenience (4 items; example item 5: “Running out of battery in my smartphone would scare me,” Alpha = 0.94).

#### 2.3.3 Mobile phone involvement questionnaire

Mobile addiction was measured using the Mobile Phone Involvement Questionnaire (MPIQ; Walsh et al., 2010), which is based on Brown’s (1997) behavioral addiction components and qualitative descriptions of mobile phone behavior (Walsh et al., 2008). It assesses withdrawal, cognitive and behavioral salience, euphoria, loss of control, relapse and reinstatement, conflict with other activities, and interpersonal conflict. The MPIQ is a one-dimensional 8-item measure of mobile phone involvement scored on a 7-point Likert scale ranging from 1 (strongly disagree) to 7 (strongly agree). Each item represents the degree to which interacting with a mobile phone is perceived as integral to everyday life. Example items are “I often use my smartphone for no particular reason” and “The thought of being without my smartphone makes me feel distressed.” It had a Cronbach’s alpha of 0.85.

Although the NMP-Q and MPIQ assess related aspects of problematic smartphone use, they target different underlying processes: anxiety-driven reactions versus behavioral dependence. This distinction was taken into account when interpreting their associations.

### 2.4 Analysis

Preliminary analyses were computed: descriptive analysis and correlations. Three hierarchical multiple regression analyses were then calculated to test Hypothesis 1 and Hypothesis 4. A sensitivity analysis was conducted to determine the required sample size to detect significant interaction effects in the moderation models. Results showed that the study’s sample size (*N* = 366) was sufficient to detect medium to large interaction effects with a power of 0.80, assuming a significance level of 0.05. Following Cohen and Cohen (1983), the lower-order variables were introduced first and the higher-order terms afterwards. Control variables were entered in step 1: sex. In step 2, the predictor variables (mobile addiction and age) were introduced, then in step 3, the interaction terms between variables were introduced. We used centered scores to overcome any possible problem of multicollinearity and to maximize interpretability. In addition, a graphical representation was performed to better understand the nature of the interactions (Jaccard et al., 1990). Finally, the ANOVAs were computed to examine the second and third hypotheses. The age measure was codified as a categorical variable with four response options: under 30 years of age, 31–40, 41–50, and over 51 years of age. The Bonferroni post-test was also computed to clarify the differences in results when comparing age groups. Age was analysed both as a continuous and as a categorical variable depending on the analytical objective. In the regression and moderation analyses, age was treated as a continuous variable to preserve the full variability of the data and maximize statistical power when testing interaction effects. For the ANOVA analyses, age was categorized into four groups (≤30, 31–40, 41–50, and ≥51 years) to facilitate the interpretation of potential non-linear differences across meaningful developmental stages, consistent with prior research. In addition, effect sizes were calculated and reported to improve the interpretability of the findings. Specifically, Cohen’s f^2^ was considered for regression models and partial eta squared (ηp^2^) for ANOVA analyses.

## 3 Results

### 3.1 Descriptive statistics

Table 1 presents the means, standard deviations, and correlations between variables. Most of the variables were significantly related. The correlations ranged from 0.10 to 0.65. Age was negatively related to both nomophobia and mobile addiction, while mobile addiction was positively related to nomophobia.

**Table 1:** Descriptive analysis (mean and standard deviation) and correlations.

	Mean	SD	1	2	3	4	5	6	7
1. Gender	1.550	0.498	-						
2. Mobile addiction	2.308	0.8259	0.206^**^	-					
3. Age	40.430	13.318	−0.121^*^	−0.406^**^					
4. Fear of not being able to communicate by smartphone	2.830	0.999	0.240^**^	0.534^**^	−0.107^*^				
5. Fear of losing the connectedness that smartphones provide	1.937	0.885	0.094	0.591^**^	−0.114^*^	0.617^**^			
6. Fear of not being able to access information	3.062	1.022	0.079	0.571^**^	−0.226^**^	0.537^**^	0.495^**^		
7. Fear of giving up convenience	2.572	0.977	0.222^**^	0.650^**^	−0.211^**^	0.710^**^	0.625^**^	0.632^**^	-

*p < 0.05; **p < 0.001.

### 3.2 Regression analyses

Table 2 presents the regression results. These support the positive relationships between mobile addiction and nomophobia: fear of not being able to communicate (H1a), losing the connectedness that smartphones provide (H1b), not being able to access information (H1c), and giving up convenience (H1d). Thus, people with mobile addiction also experience nomophobia in its four dimensions.

**Table 2:** Hierarchical multiple regression analysis of mobile addiction and age in predicting nomophobia.

	Fear of not being able to communicate	Fear of losing the connectedness that smartphones provide	Fear of not being able to access information	Fear of giving up convenience
Step 1				
Sex (1 man; 2 women)	0.24**	0.09	0.08	0.22**
Step 2				
Mobile addiction	0.56**	0.66**	0.58**	0.66**
Age	0.14**	0.15**	0.01	0.07
Step 3				
Mobile addiction*age	0.09*	0.03	0.10**	0.08*
R^2^	0.32*	0.36	0.33**	0.43*
R^2^ change step 1	0.05**	0.01**	0.01	0.04**
R^2^ change step 2	0.26**	0.35**	0.32**	0.38**
R^2^ change step 3	0.01*	0.00	0.01**	0.01*

*p ≤ 0.05 **p ≤ 0.01 ***p ≤ 0.1 two-tailed. The values are the standardized regression coefficients from the significant final stage of the regression analysis.

### 3.3 Age differences in nomophobia and mobile addiction

Table 3 presents the ANOVA results, including effect sizes (partial eta squared, ηp^2^). These partially support Hypothesis 2 insofar as they show significant differences in nomophobia levels in function of age (H2c, fear of not being able to access information, and H2d, fear of giving up convenience). However, they do not show any differences in connection with the fear of not being able to communicate by smartphone (H2a) and losing the connectedness that smartphones provide (H2b). The Bonferroni test indicated that people under 30 years of age presented greater fear of not being able to access information and of giving up convenience than those aged between 41 and 50 years and those 51 years and over. However, there were no differences in the levels of fear of not being able to access information and of giving up convenience between people under 30 years of age and those between 31 and 40 years.

**Table 3:** ANOVA results: nomophobia and mobile addiction differences in the function of age.

Dimensions	F	df	*p*-value
Fear of not being able to communicate	3.247	1	0.356
Fear of losing the connectedness that smartphones provide	3.438	1	0.223
Fear of not being able to access information	17.547	1	0.001
Fear of giving up convenience	14.196	1	0.002
Mobile addiction	37.585	1	0.000

Age was a categorical variable: (1) up to 30 years old, (2) 31–40 years old, (3) 41–50 years old, and (4) 51 years old and over. Effect sizes are reported as partial eta squared (ηp^2^).

Hypothesis 3, which states that there will be significant differences in mobile addiction between older and younger people, was supported by the results. The ANOVA showed significant differences in mobile addiction between age groups. The Bonferroni test showed that people under 30 years of age presented higher levels of mobile addiction than those between 31 and 40 years, those between 41 and 50 years, and those 51 years and over. Indeed, the results also showed that people in the age range from 31 to 40 presented higher levels of mobile addiction than those aged 51 years and over (see Table 4).

**Table 4:** Bonferroni post hoc test: multiple comparisons.

Dependent variable	(I) Age group	(J) Age group	Mean difference (I-J)	*p*-value
Fear of not being able to access information	1	2	0.145	1.00
		3.00	0.442	0.012
		4.00	0.526	0.002
	2.00	1.00	−0.145	1.00
		3.00	0.297	0.361
		4.00	0.380	0.101
	3.00	1.00	−0.442	0.012
		2.00	−0.297	0.361
		4.00	0.083	1.000
	4.00	1.00	−0.526	0.002
		2.00	−0.380	0.101
		3.00	−0.083	1.000
Fear of giving up convenience	1.00	2.00	0.070	1.000
		3.00	0.430	0.010
		4.00	0.412	0.017
	2.00	1.00	−0.070	1.000
		3.00	0.359	0.107
		4.00	0.342	0.150
	3.00	1.00	−0.430	0.010
		2.00	−0.359	0.107
		4.00	−0.017	1.000
	4.00	1.00	−0.412	0.017
		2.00	−0.342	0.150
		3.00	0.017	1.000
Mobile addiction	1.00	2.00	0.428	0.003
		3.00	0.606	0.000
		4.00	0.845	0.000
	2.00	1.00	−0.428	0.003
		3.00	0.178	0.828
		4.00	0.416	0.004
	3.00	1.00	−0.606	0.000
		2.00	−0.178	0.828
		4.00	0.238	0.166
	4.00	1.00	−0.845	0.000
		2.00	−0.416	0.004
		3.00	−0.238	0.166

Age was a categorical variable: (1) up to 30 years old, (2) 31–40 years old, (3) 41–50 years old, and (4) 51 years old and over.

### 3.4 Moderation analysis: age as a moderator

The regression results also supported Hypothesis 4 (Table 2). They showed that age moderated the relationship between mobile addiction and nomophobia, but for only three of the dimensions of nomophobia: fear of not being able to communicate by smartphone (H4a), of not being able to access information (H4c), and of giving up convenience (H4d). To better understand the nature of these interactions, different graphical representations were performed. The moderation effect of age on the relationship between mobile addiction and nomophobia was significant (b = 0.25, *p* < 0.05, f^2^ = 0.04), indicating a medium effect size and providing confidence in the robustness of our findings despite the sample size.

**Figure 1:**
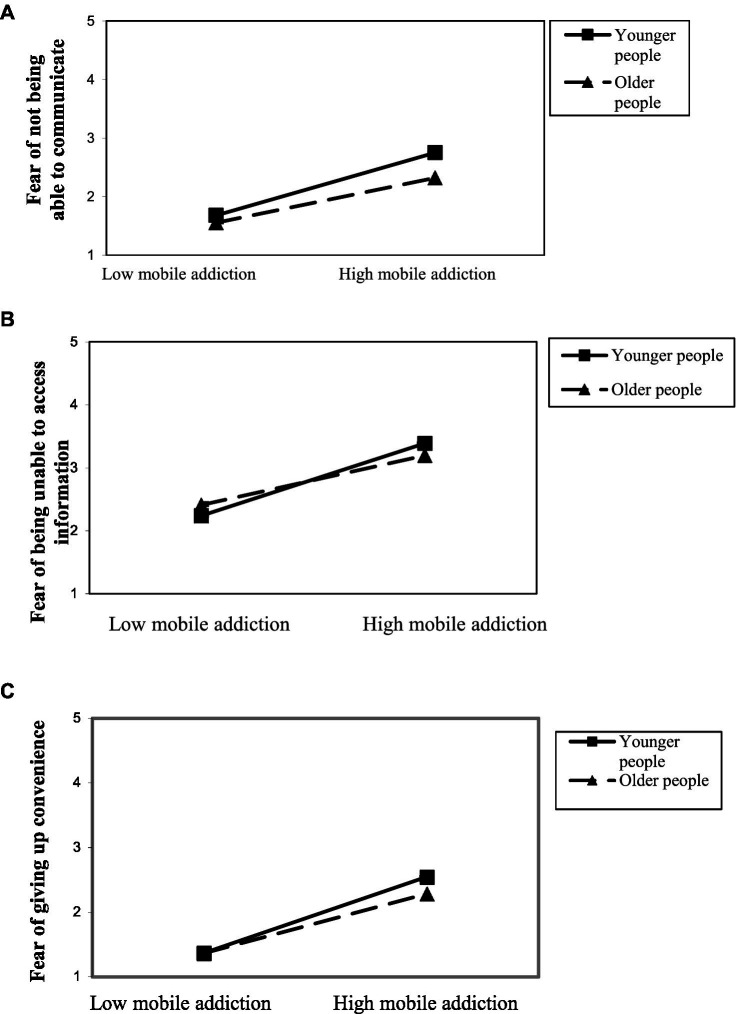
Interaction of mobile addiction and age in predicting **(A)** fear of not being able to communicate, **(B)** fear of not being able to access information, and **(C)** fear of giving up convenience.

Figure 1A plots the interaction between mobile addiction and age to predict the fear of not being able to communicate by smartphone (H4a). The greater the mobile addiction, the greater the fear of not being able to communicate. The slope is steeper for younger people than for older people, which means that younger people with mobile addiction experience greater fear in this area.

Figure 1B plots the interaction between mobile addiction and age to predict the fear of not being able to access information (H4c). The greater the mobile addiction, the greater the fear of not being able to access information. The slope was steeper for younger people than for older people. Therefore, younger people with mobile addiction experienced greater fear of not being able to access information than older people.

Figure 1C shows a graphical representation of the interaction between mobile addiction and age to predict the fear of giving up convenience (H4d). The greater the mobile addiction experienced, the greater the fear of giving up convenience. The slope is steeper for younger people than for older people, and thus, younger people with mobile addiction experience greater fear of giving up convenience than older people.

## 4 Discussion

The early and unstoppable incorporation of the mobile phone into everyday life is undoubtedly the most forceful example of the technological onslaught that has taken place over the last two decades. The mobile phone has become an essential resource in people’s lives, but it has also had some negative consequences for health, such as the emergence of two problematic smartphone-related phenomena: mobile addiction and nomophobia. These two are conceptually related but represent distinct psychological processes. Nomophobia refers primarily to anxiety-driven reactions associated with the perceived unavailability of the smartphone (e.g., fear of disconnection and inability to access information), whereas smartphone addiction reflects behavioral dependence characterized by salience, withdrawal, loss of control, and relapse in mobile phone use (King et al., 2013; Yildirim and Correia, 2015; Walsh et al., 2010). Consistent with prior literature, the significant associations observed between the constructs support their relatedness, while their differentiated predictive and moderating roles suggest they function as distinct but interconnected phenomena (Tran, 2016; Kaviani et al., 2020). This study aimed to clarify the conceptualization of nomophobia compared to mobile addiction and to examine the relationship between the two constructs, along with the role that age may play in these constructs and their relationship to them. These findings should be interpreted as associative rather than causal, given the cross-sectional nature of the data.

The first contribution of this study was to show the positive association between mobile addiction and the different dimensions of nomophobia [fear of not being able to communicate by smartphone (H1a), of losing the connectedness that smartphones provide (H1b), of not being able to access information (H1c), and of giving up convenience (H1d)]. Greater mobile addiction was associated with greater nomophobia across all dimensions. These results are congruent with previous literature showing an association between mobile addiction and nomophobia (e.g., Durak, 2019; Davie and Hilber, 2017). These findings extend previous work (e.g., Ren et al., 2024) by demonstrating that the association between mobile addiction and nomophobia is not uniform across individuals but varies according to age, highlighting the importance of considering developmental factors when examining problematic smartphone-related behaviors.

The second contribution was to provide additional evidence on the differences in levels of nomophobia between different age stages. Our results show that the levels of fear of not being able to access information and of giving up convenience were higher among younger than older people. However, there were no differences between age groups in the dimensions of fear of not being able to communicate by smartphone and of losing the connectedness that smartphones provide. These results are congruent with previous literature that has shown the association between age and nomophobia (Yildirim and Correia, 2015; Moreno-Guerrero et al., 2020) and also with those studies that showed non-significant associations involving the fear of not being able to communicate (e.g., Moreno-Guerrero et al., 2020). According to our results, we can conclude that the age variable is critical in nomophobia.

The third contribution was to provide additional evidence regarding the differences in mobile addiction between different age stages. Our results show that there are significant differences in levels of mobile addiction between the oldest and youngest populations. Younger people are more likely to experience greater levels of mobile addiction compared to older people. These findings extend prior research by demonstrating that age not only affects the levels of nomophobia and mobile addiction but also moderates their relationship. Studies, such as the one by Lee et al. (2018), have emphasized the importance of considering age as a moderating factor in understanding the developmental implications of smartphone use. In contrast to previous research, our study further explores the complex, multi-dimensional nature of nomophobia and mobile addiction and how these constructs vary in strength depending on developmental stages. These results are congruent with previous literature on mobile addiction (e.g., Moreno-Guerrero et al., 2020; Darvishi et al., 2019).

The fourth contribution showed the moderating role of age in the relationship between mobile addiction and nomophobia. The interaction between mobile addiction and age could explain three of the dimensions of nomophobia: fear of not being able to communicate, fear of not being able to access information, and fear of giving up convenience. These results are in line with the literature on mobile addiction and nomophobia (e.g., Moreno-Guerrero et al., 2020; Yildirim and Correia, 2015).

### 4.1 Limitations

Although these results contain much of interest, they should be interpreted bearing in mind the potential limitations of the study. One is that the causal relationships between variables cannot be inferred because a cross-sectional design was used. There are few longitudinal studies in the nomophobia literature that test causal relationships over time, so further research is needed in this area. Another possible limitation is that all our variables were assessed by self-reported questionnaires. The results may therefore be influenced by common method variance. Other methods could usefully be applied in future research to provide further evidence on the relationships found here.

### 4.2 Theoretical and practical implications

These results carry several theoretical and practical implications. Our study contributes to the literature by examining the relationship between mobile addiction and nomophobia, plus the role that age can play in these psychological disorders and their relationship to them. Regarding practical implications, the study could point to the need for specific interventions to be designed for nomophobia and mobile addiction, oriented toward young people and their needs. It may thus be possible to promote a life more balanced between virtual and direct interactions to prevent the emergence of mobile addiction and nomophobia.

### 4.3 Future research

Most of the research has focused on the psychological and individual characteristics that can determine nomophobia and mobile addiction. Future research is therefore needed to investigate the social factors that influence the development of addictive behaviors toward the smartphone and those phobias generated by being permanently connected. More research is also necessary to explore the potential factors that could affect the relationship between mobile addiction and nomophobia, other than age. Examples of these critical intervening factors could include both individual characteristics (e.g., gender, education level, personality, and socioeconomic level) and social factors (e.g., social skills, family, and social context). Although nomophobia and mobile addiction share common features and may co-occur, the present findings provide support for their conceptual differentiation. Future research should further examine their discriminant validity using advanced psychometric approaches such as confirmatory factor analyses, bifactor models, or longitudinal designs to better establish their independence over time.

## 5 Conclusion

This research has shown that mobile addiction and nomophobia are different but related psychological constructs. Mobile addiction is a determining factor for nomophobia, and therefore, people who present the former are likely to experience the latter. The research has also shown that age is a critical factor for both nomophobia and mobile addiction. Younger people were found to have higher levels of mobile addiction compared to older people. However, the effect of age on nomophobia varies. The only differences in the function of age were found in the dimensions of fear of not being able to access information and of giving up convenience. Finally, age was seen to be critical in the relationship between mobile addiction and nomophobia insofar as it moderated this association by showing that younger people with mobile addiction present higher levels of nomophobia.

## Data Availability

The raw data supporting the conclusions of this article will be made available by the authors, without undue reservation.
